# Emerging insights into the pathogenesis and therapeutic strategies for vascular endothelial injury-associated diseases: focus on mitochondrial dysfunction

**DOI:** 10.1007/s10456-024-09938-4

**Published:** 2024-07-26

**Authors:** Boxian Pang, Guangtong Dong, Tieliang Pang, Xinyao Sun, Xin Liu, Yifeng Nie, Xing Chang

**Affiliations:** 1 Beijing University of Chinese Medicine, Beijing, China; 2 CAS Center for Excellence in Nanoscience, National Center for Nanoscience and Technology, Beijing, China; 3 Beijing Anding hospital, Capital Medical University, Beijing, China; 4 Bioscience Department, University of Nottingham, Nottingham, UK; 5 Guang’anmen Hospital, China Academy of Chinese Medical Sciences, 5 Beixiagge, Xicheng District, Beijing, China

**Keywords:** Vascular endothelial injury, Mitochondria dysfunction, Clinical disease, Pathogenesis, Therapeutic strategies

## Abstract

As a vital component of blood vessels, endothelial cells play a key role in maintaining overall physiological function by residing between circulating blood and semi-solid tissue. Various stress stimuli can induce endothelial injury, leading to the onset of corresponding diseases in the body. In recent years, the importance of mitochondria in vascular endothelial injury has become increasingly apparent. Mitochondria, as the primary site of cellular aerobic respiration and the organelle for “energy information transfer,” can detect endothelial cell damage by integrating and receiving various external stress signals. The generation of reactive oxygen species (ROS) and mitochondrial dysfunction often determine the evolution of endothelial cell injury towards necrosis or apoptosis. Therefore, mitochondria are closely associated with endothelial cell function, helping to determine the progression of clinical diseases. This article comprehensively reviews the interconnection and pathogenesis of mitochondrial-induced vascular endothelial cell injury in cardiovascular diseases, renal diseases, pulmonary-related diseases, cerebrovascular diseases, and microvascular diseases associated with diabetes. Corresponding therapeutic approaches are also provided. Additionally, strategies for using clinical drugs to treat vascular endothelial injury-based diseases are discussed, aiming to offer new insights and treatment options for the clinical diagnosis of related vascular injuries.

## Introduction

Endothelial cells (ECs), constituting the flattened endothelial tissue covering the inner layers of the heart, blood vessels, and lymphatic vessels, serve as both mechanical and biological barriers to blood flow [1]. The main functions of ECs include conduit transportation and barrier protection, nutrient support, and regulation of vascular tone as a paracrine organ. These three functions are synergistic [2]. Under normal conditions, the impermeable endothelium acts as a selective barrier, facilitating nutrient transport. However, endothelial barrier dysfunction can lead to extravasation of nutrients, leukocytes and platelets [2]. Furthermore, under local stimulation, ECs secrete potent vasoconstrictors, endothelin and leukotriene C4, and vasodilators nitric oxide (NO) and prostacyclin (PGI2), impacting vascular smooth muscle tone [3]. Past studies have demonstrated the crucial role of ECs in maintaining vascular homeostasis. EC loss leads to vascular dysfunction, contributing to early events in atherosclerosis, hypertension, and diabetes [3]. In recent years, there has been increasing recognition of the importance of mitochondria in vascular endothelium damage.

Mitochondria are the main sites of cellular aerobic respiration. Reactive oxygen species (ROS) production by mitochondria and mitochondrial dysfunction often determine the evolution of EC injury toward necrosis or apoptosis [4]. Therefore, mitochondrial homeostasis determines whether ECs are normal or injured. Previous research has established the importance of mitochondrial dynamics and mitophagy in endothelial mitochondrial homeostasis [5–8]. Mitochondrial dynamics involve mitochondrial fission and fusion. Mitochondrial fission permits isolation and subsequent degradation of damaged depolarized mitochondria, whereas mitochondrial fusion dilutes potentially harmful components [9]. Mitochondrial division is mainly controlled by dynamin-related protein 1 (Drp1) and fission 1 protein (Fis1); whereas fusion is controlled by mitofusin 1 and 2 (Mfn 1 and 2) [10]. Abnormal expression of control proteins leads to abnormal mitochondrial division and fusion. Aberrant mitochondrial fission leads to insufficient mitochondrial numbers, hindering EC metabolism and proliferation [11]. On the other hand, abnormal mitochondrial fusion results in an inability to exchange genetic material and metabolic substrates between different mitochondria, thereby compromising mitochondrial stability and integrity [12]. Mitophagy, a lysosome-mediated process of mitochondrial self-clearance, is essential for maintaining the stability and function of the entire mitochondrial network [13]. Dysregulated mitophagy fails to degrade damaged mitochondria, further destabilizing mitochondrial structure and function. Thus, mitochondrial dysfunction-induced endothelial injury may represent an early event in many vascular-related diseases.

This article comprehensively reviews the pathogenesis of mitochondrial-induced vascular endothelial injury in cardiovascular diseases, pulmonary vascular diseases, kidney-related vascular diseases, cerebrovascular diseases, and microvascular diseases associated with diabetes. Furthermore, it provides corresponding therapeutic strategies, including the use of clinical drugs, aiming to offer novel insights and treatment approaches for the clinical diagnosis of related vascular injuries.

## Cardiovascular diseases

**Fig. 1:**
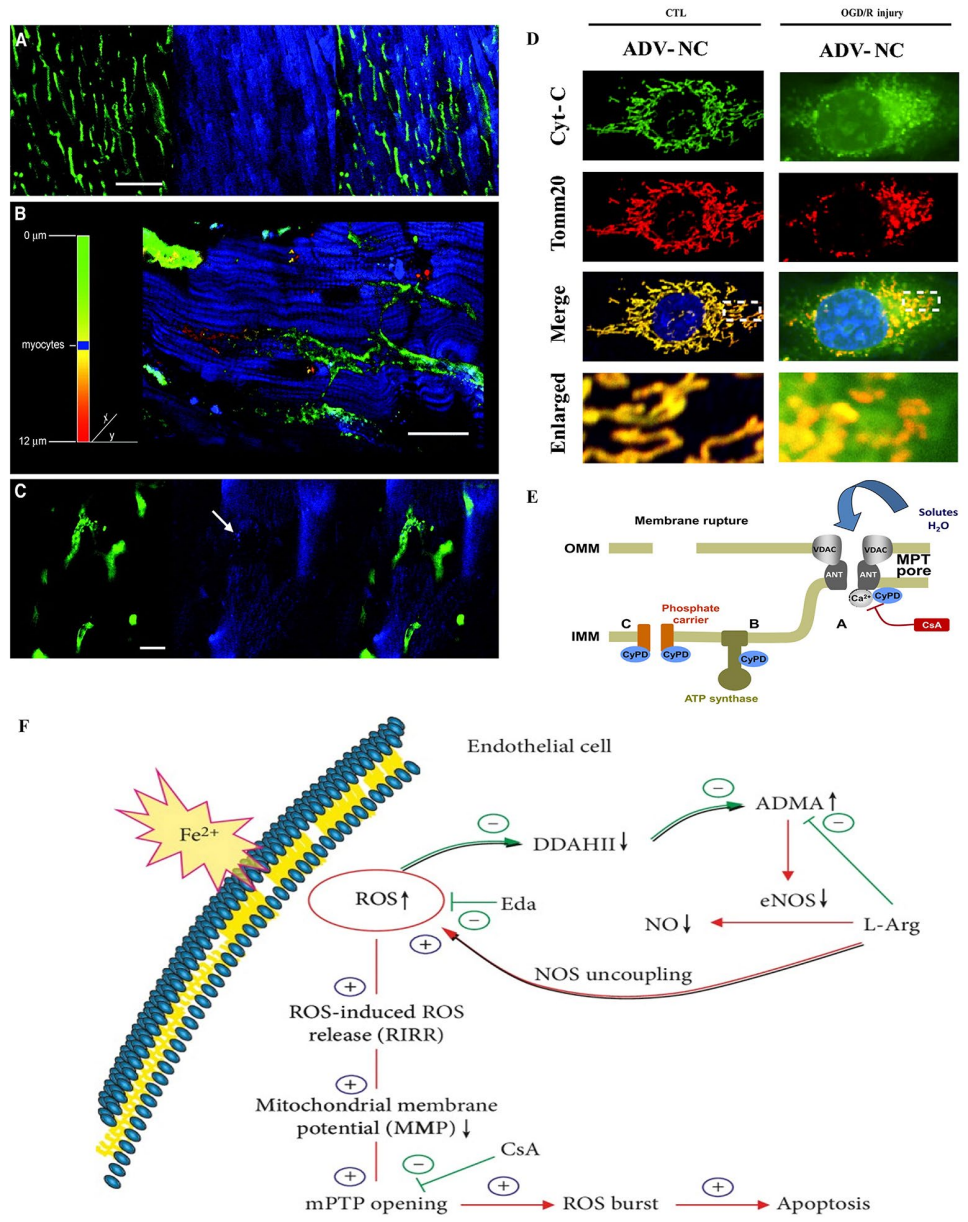
Mitochondria affect endothelial cell function during normal physiological processes. (**A**-**C**), Fluorescence living cell images of rat left ventricular myocardium. Endothelial cells were stained with fluorescent-labeled lectin (green) and multiphoton microscopy showed that endothelial cells meandered around and around cardiomyocytes (blue), playing an important role as “conduits” and also regulating mitochondrial activity in cardiomyocytes. (**A**), Low magnification illustrates the degree of myocardial vascularization. Scale bars: 100 μm. (**B**), Different colors from green to red represent different depths of endothelium and cardiac myocytes in blue shown at 6 μm depth. Scale bars: 20 μm. (**C**), Fluorescence images at high magnification, NAD(P)H autofluorescence could be detected from endothelialmitochondria (arrow). Scale bars: 10 μm. Copyright 2007, Reprinted with from permission from Wolters Kluwer Health, Inc [2]. **D** Under conditions of oxygen-glucose deprivation/reoxygenation (OGD/R) injury, cardiac microvascular ECs (CMECs) undergo mitochondrial fission leading to cytochrome C release, resulting in activation of mitochondria-dependent apoptotic pathways. The analysis of colocalization between mitochondria and cytochrome C was performed by immunostaining of cytochrome C (green) and Tomm20 (red). Cyt-C: cytochrome C. Scale bars: 10 μm. Copyright 2021, Reprinted with from permission from Springer Nature Publishing Group [14]. (**E**) Mitochondrial Ca^2+^ accumulation can trigger the opening of high-conductance pores and permeability transition in the inner mitochondrial membrane (IMM). Copyright 2015, Reprinted with from permission from Elsevier B.V. [15]. (**F**) Diagram showing the possible mechanisms of iron overload induced VEC damage. Excessive free iron ions produce excessive ROS in the cytoplasm that inhibit DDAHII and accumulate ADMA. ADMA not only competitively inhibits eNOS activity, but also induces the decoupling of eNOS to produce more ROS, which leads to a vicious cycle. In addition, excessive ROS enter mitochondria and activate RIRR mechanism, which these two cycles together induce mitochondrial dysfunction and VEC damage. Copyright 2019, Reprinted with from permission from Hindawi Publishing Group [16]

Cardiac endothelial cells are one of the main cellular components of the heart, and there is at least one capillary in the vicinity of each cardiomyocyte (Fig. 1A), snaking around each cardiomyocyte (Fig. 1B) and playing an important “plumbing” role (Fig. 1C) [2]. Endothelial dysfunction has been identified as a major mediator of cardiovascular diseases and is closely related to mitochondrial abnormalities that regulate the structure and function of endothelial cells themselves. Under conditions of oxygen-glucose deprivation/reoxygenation (OGD/R) injury, cardiac microvascular ECs (CMECs) undergo mitochondrial fission leading to cytochrome C release (cytochrome C is released from the mitochondria into the cytoplasm of the CMECs) resulting in activation of mitochondria-dependent apoptotic pathways [14]. This was evidenced by the reduced co-localization of mitochondria and cytochrome C (Fig. 1D). Meanwhile, cardiovascular illness may result in endothelial mitochondrial dysfunction, which harms endothelial cells and exacerbates cardiovascular conditions. Previous studies have shown that mitochondrial damage after myocardial infarction induces vascular inflammation further impairing cardiovascular health [17]. Acute myocardial ischemia results in oxidative damage to ECs’ mitochondria, which subsequently activate GABARAPL1-induced NLRP3 Inflammasomes via an Autophagic-Exosome Manner. The NLRP3 Inflammasomes stimulus induces the proliferation of monocytes and neutrophils, which ultimately leads to vascular inflammation [17].

Deacetylase sirtuin, ionic homeostasis, and several other factors have important effects on cardiovascular endothelial cell mitochondria. This section summarizes the mechanisms of influence and treatment related to these factors.

### Deacetylase sirtuin

The Sirtuin family is the first discovered class III HDAC. They all possess a highly conserved nicotinamide adenine dinucleotide (NAD+) binding domain and a catalytic domain, while different N-terminal and C-terminal structures allow them to bind to different substrates [18]. SIRT1 and SIRT3 are two well-characterized cardioprotective isoforms [19]. SIRT1, as the most extensively studied star molecule in the family, participates in various mechanisms including metabolism, immune response, and aging regulation. It regulates nuclear-encoded proteins involved in mitochondrial biogenesis and can directly control the expression of mitochondrial genes. Studies have shown that overexpression of SIRT1 can prevent cardiomyocytes from suffering myocardial I/R injury-induced cell death and oxidative stress damage [20]. Furthermore, activation of the adenosine 5’-monophosphate (AMP)-activated protein kinase (AMPK)/SIRT1/peroxisome proliferator-activated receptor γ coactivator 1-alpha (PGC-1α) pathway is essential in mitigating mitochondrial oxidative stress damage [21]. The upstream regulatory protein of SIRT, AMPK, has been reported to be activated during myocardial I/R injury, exerting protective effects by regulating various metabolic pathways, including mitochondrial function [22, 23]. Specifically, AMPK activation induces SIRT1 to regulate PGC1α activity, thereby reducing mitochondrial oxidative stress damage [22]. The therapeutic drug, Xinmai’an tablets, composed of six traditional Chinese medicines, can reduce myocardial infarct size, alleviate myocardial and endothelial injury, and protect the heart by acting through the AMPK/SIRT1/PGC-1 pathway [21]. p66Shc stimulates mitochondrial ROS production through its oxidoreductase activity, while SIRT 1 negatively regulates p66Shc expression at the transcriptional level to suppress the effects of ROS, protecting ECs from oxidative damage [24]. The specific mechanism involves SIRT1 binding to the p66Shc promoter, leading to histone H3 deacetylation, thereby attenuating p66Shc transcription and translation [24]. SIRT3 serves as a primary regulator of mitochondrial energy metabolism, influencing mitochondrial respiratory function through cytochrome C (CytC) [18]. Sirt3 deficiency may result in mitochondrial protein hyperacetylation, promoting endothelial dysfunction, increasing smooth muscle cell hypertrophy, inducing vascular inflammation, and triggering age-dependent hypertension [25]. Animal experiments have shown that decreased expression of Sirt3 leads to vascular dysfunction and hypertension [26]. Recent studies have found lower levels of the mitochondrial deacetylase, sirtuin (Sirt) 3, in hypertensive patients compared to normal individuals [27].

### Ionic homeostasis

Disruption of ionic homeostasis can lead to mitochondrial dysfunction. In recent years, increasing evidence suggests that mitochondrial dysfunction caused by mitochondrial calcium overload leads to endothelial dysfunction and cardiomyocyte apoptosis [28–30]. Mitochondrial Ca^2+^ accumulation can trigger the opening of high-conductance pores in the inner mitochondrial membrane (IMM) (Fig. 1E). This phenomenon is known as mitochondrial permeability transition (MPT). Subsequently, there is mitochondrial swelling and rupture of the mitochondrial membrane, leading to the release of mitochondrial proteins including cytochrome c into the cytosol. Angiotensin II (Ang-II) stimulates vascular endothelial cells (VECs) to secrete endothelin (ET), activating intracellular signaling pathways that open calcium ionic channels [31]. Simultaneously, Ang-II reduces the activity of Na+-K + ATPase, inhibiting the function of the Na^+^-K^+^ pump, ultimately leading to the opening of calcium ion channels and massive influx of calcium ions, resulting in calcium overload [31]. Li’s study demonstrates that tea polyphenols protect VECs from calcium overload-induced damage by enhancing mitochondrial membrane potential (MMP) and reducing endothelin production and intracellular calcium levels induced by Ang-II [31]. Another study by Li reported the protective effect of dihydromyricetin against vascular endothelial cell injury induced by angiotensin II [32]. The specific mechanism involves dihydromyricetin increasing MMP levels, reducing Ca^2+^ levels, and lowering AngII levels in endothelial tissue, thereby protecting VEC mitochondria and shielding VEC from AngII-induced damage [32]. In addition to Ang-II-mediated calcium overload, hyperuricemia also leads to mitochondrial calcium overload via the mitochondrial Na^+^/Ca^2+^ exchanger [33]. In terms of treatment, Li et al. reported the inhibitory mechanism of Histidine triad (HIT) nucleotide-binding protein 2 (HINT2) on mitochondrial calcium overload in CMECs [14]. HINT2 can directly interact with the mitochondrial calcium uniporter (MCU) complex in CMECs, inhibiting mitochondrial calcium overload [14]. This enhances cardiomyocyte survival rates and protects cardiac function under ischemic conditions.

In addition to calcium overload, mitochondrial iron overload can lead to mitochondrial dysfunction. Iron overload leads to mitochondrial dysfunction in endothelial cells, thereby damaging VECs. He et al. [16]. reported the mechanism by which iron overload damages endothelial mitochondria via the ROS/ADMA/DDAHII/eNOS/NO pathway (Fig. 1F). On the one hand, excessive free iron ions in the cytoplasm generate an overload of reactive oxygen species (ROS), inhibiting DDAHII and accumulating ADMA. ADMA not only competitively inhibits eNOS activity, reducing NO synthesis, but also induces eNOS uncoupling, generating more ROS, thereby establishing a vicious cycle of ROS production [16]. On the other hand, excessive ROS enter mitochondria, weakening mitochondrial membrane potential (MMP), opening the mitochondrial permeability transition pore (mPTP), activating the RIRR mechanism, forming another vicious cycle [16]. Targeting the ROS/ADMA/DDAHII/eNOS/NO pathway, Chen et al. found that Luteoloside could target endothelial cell mitochondria via the ROS/ADMA/DDAHII/eNOS/NO pathway to protect the vascular endothelium from iron overload injury [34]. Mitoferrin 2 (Mfrn2) is an iron transport protein on the inner membrane of mitochondria. Studies have shown that TNF-α increases the binding of 14-3-3 epsilon (ε) to Mfrn2, preventing the degradation of Mfrn2, leading to mitochondrial iron overload in ECs [35]. Silencing the Mfrn2 gene can inhibit TNF-α-induced mitochondrial iron overload, thereby stabilizing mitochondrial membrane potential and improving mitochondrial function [35]. The therapeutic drug, isoliquiritigenin, is a compound similar to resveratrol, possessing potent antioxidant capabilities and cardiovascular protective effects [36]. Research by Chen et al. suggests that isoliquiritigenin inhibits mitochondrial iron toxicity through the PRDX2-MFN2-ACSl4 pathway, beneficially protecting the cardiac microvasculature in diabetic patients [36].

Copper also plays a crucial role in maintaining mitochondrial function. Research indicates that homocysteine disrupts copper homeostasis in endothelial cells. Elevated homocysteine levels decrease intracellular copper concentration and lead to redistribution of copper among different molecular weight fractions, limiting its availability in higher molecular weight fractions [37]. This redistribution restricts effective utilization of copper by key molecules, resulting in reduced activity of cytochrome c oxidase (CCO), an essential component of the mitochondrial respiratory chain [37]. Additionally, levels of the copper chaperone protein COX17, closely associated with CCO, decline accordingly [37]. COX17 is responsible for delivering copper to CCO [38]. These changes ultimately lead to collapse of mitochondrial membrane potential, impairing normal mitochondrial function and causing damage to endothelial cells [37].

Zinc is commonly regarded as an antioxidant with cellular protective properties [39]. Previous studies have demonstrated the critical importance of zinc in endothelial integrity, where zinc deficiency can severely compromise endothelial barrier function [40]. Zinc ions can protect endothelial cells by inhibiting the activation of caspase-3 subunits [39]. However, recent research suggests that zinc ions not only have protective effects but can also impair mitochondrial function and induce mitochondrial autophagy in cardiomyocytes under conditions of overload [41]. Zinc ion overload leads to decreased mitochondrial membrane potential, reduced expression of Mfn2, increased levels of reactive oxygen species (ROS) in both the cytoplasm and mitochondria, resulting in mitochondrial dysfunction [41]. Zinc overload induces mitochondrial autophagy through two pathways: activation of the PINK1/Parkin signaling pathway and Mfn2-mediated ROS induction [41].

### Other factors

Activation of some hormone receptors also induces mitochondrial disorders leading to cardiovascular disease. Activation of histamine H2 receptors can elevate levels of phospho-extracellular signal-regulated protein kinases 1 and 2 (p-ERK1/2), Bcl-2-associated X protein (Bax), phospho-death associated protein kinase (p-DAPK2), and caspase 3, promoting myocardial mitochondrial dysfunction and increasing cardiac endothelial permeability, thus exacerbating myocardial ischemia/reperfusion (I/R) injury [42]. Activation of mineralocorticoid receptors initiates inflammation and fibrosis in the heart by increasing nicotinamide adenine dinucleotide phosphate (NADPH) oxidase and mitochondrial ROS production [43].

Additionally, tissue damage frequently occurs during myocardial ischemia/reperfusion (I/R) [44]. Endothelial cells contribute to this tissue damage through the coordination of the complement cascade. Studies have shown that endothelial cell mitochondria may be a source of activated complement molecules during myocardial I/R injury [44]. The mitochondria of endothelial cells may release certain molecules that can bind to C1q molecules in the complement cascade, thereby triggering the activation of the classical complement pathway [44].

**Fig. 2:**
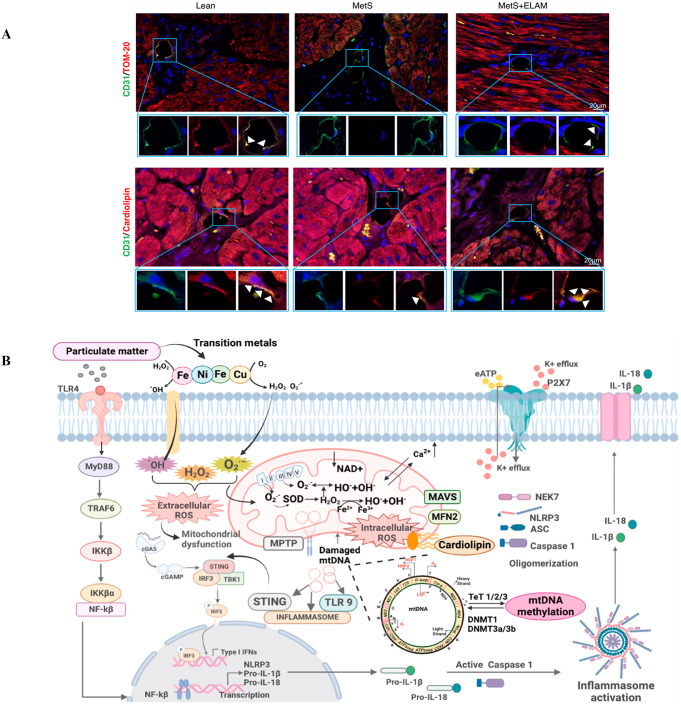
Mitochondrial abnormalities cause damage to the cardiovascular endothelium. (**A**), Metabolic syndrome (MetS) could induce mitochondrial damage in endothelial cells and Elamipretide (ELAM) restores endothelial cell cardiolipin content. Double immunofluorescent staining of endothelial marker CD31 (green) and mitochondrial markers outer membrane transferase (TOM)-20(red) and mitochondrial inner membrane phospholipids (red) shows reduced expression of endothelial mitochondria and cardiolipin (yellow combined) in metabolic syndrome (METS) and normal in ELAM treated animal models. Copyright 2018, Reprinted with from permission from American Physiological Society [46]. (**B**), Mechanism and process of mitochondrial dysfunction caused by particulate matter in cardiovascular diseases. The transition metals present on the surface of particulate matter are capable of generating both intracellular and extracellular ROS which causes mitochondrial dysfunction. PM: Particulate Matter, TLR4:Toll-like receptor 4, MyD88: Myeloid differentiation primary response Protein 88, TRAF6:TNF receptor associated factor 6, IKKB: Inhibitor of kappa B kinase, NF-kB: Nuclear factor kappa B, mtDNA: Mitochondrial DNA, ACS: Acute coronary syndrome, ROS: Reactive oxygen species, cGAS: Cyclic GMP-AMP synthase, cGAMP: Cyclic-GMP-AMP, STING: Stimulator of interferon genes, IRF3: Interferon regulatory Factor 3, TBK1:TANK Binding Kinase 1,MPTP: mitochondrial permeability transition pore, TLR9:Toll-like receptor 9, MAVS: Mitochondrial antiviral signaling protein, MFN-2: Mitofusin-2, NEK7: Never in mitosis genes related kinase 7, NLRP3: Nucleotide-binding oligomerization domain, leucine-rich repeat and pyrin domain-containing 3, DNMT1:DNA methyltransferase 1, TET1: Ten-eleven translocation, eATP: Extracellular Adenosine Triphosphate, IL-18: Interleukin-18. Copyright 2018, Reprinted with from permission from Elsevier B.V [51]

Cardiovascular and all-cause mortality rates in individuals with MetS are significantly higher than in those without MetS [45]. Animal studies have shown that MetS can cause substantial functional and structural damage to myocardial microvasculature [46]. The novel mitochondria-targeted peptide elamipretide (ELAM) targets and preserves the mitochondrial inner membrane phospholipid cardiolipin, which has been shown to have cardioprotective effects [47, 48]. Cardiolipin is an anionic phospholipid component of the mitochondrial inner membrane that plays a key role in cristae formation and mitochondrial function [49]. The peroxidation and loss of cardiolipin can activate the caspase pathway and initiate apoptosis, as well as trigger the production and release of mitochondrial reactive oxygen species [46, 50]. The study by Yuan et al. demonstrated that elamipretide (ELAM) can restore endothelial cell cardiolipin content in MetS animals (as shown in Fig. 2A), preserve coronary artery EC mitochondria, and reduce vascular damage [46].

In addition, air pollution-induced mitochondrial damage is a major risk factor for cardiovascular disease, according to a 2018 report by the World Health Organization [51]. Particulate matter (PM) in air pollution acts as pro-oxidants that generate harmful electrophilic metabolites, causing irreversible mitochondrial DNA oxidative damage, thereby damaging mitochondrial structure [52]. This structural damage can activate mitochondrial ROS production, triggering abnormal innate immune responses and promoting inflammatory reactions [53]. Furthermore, damaged mitochondria may lead to impaired oxidative phosphorylation function, triggering myocardial hypertrophy that could have serious implications for cardiovascular health [54]. The specific mechanisms associated with PM leading to mitochondrial damage are schematically shown in Fig. 2B.

Furthermore, research by Chang et al. suggests that natural antioxidants also play a crucial role in regulating mitochondrial protection of cardiovascular ECs against stress-induced damage [55]. For instance, *Panax notoginseng saponins* can reduce ROS-mediated oxidative damage, improve mitochondrial dysfunction, and inhibit cardiomyocyte apoptosis [56]; and *Ligustrazine* can ameliorate oxidative stress-induced damage in HUVECs, inhibiting the release of cytochrome C from mitochondria to the cytoplasm [57].

### Pulmonary vascular diseases

Acute lung injury (ALI)/acute respiratory distress syndrome (ARDS) is a lung disorder with limited treatment strategies and a high severity, with a mortality rate of 30–40% [58]. The mechanism of ALI/ARDS involves EC inflammation and endothelial barrier dysfunction, leading to inflammation infiltration, interstitial edema, alveolar filling, and ultimately respiratory failure [59]. Memet et al. research indicates a close association between endothelial barrier failure and increased uncoupling protein 2 (UCP2) on the mitochondrial inner membrane [60], while Dilip et al. study suggests that adiponectin (APN) deficiency can cause mitochondrial dysfunction in pulmonary ECs, leading to lung injury [61, 62]. One previous study also found that the expression level of miR-34a-5p is positively correlated with the inflammatory response of acute lung injury.The mechanism involves miR-34a causing p53 and Bax translocation to the mitochondrial compartment, disrupting mitochondrial membrane potential, and releasing cytochrome C into the cytoplasm, thereby triggering mitochondrial-mediated cascading cell apoptosis in the lungs [63]. Exposure to cigarette smoke (CS) increases the risk of acute respiratory distress syndrome (ARDS) in humans, but its mechanisms remain unclear. Wang et al. suggest that the mechanism may be related to CS-induced endothelial damage caused by mitochondrial oxidative stress and dysfunction resulting from excessive mitochondrial fission [10]. Previous literature reports that phosphorylation at the Drp1-S616 site promotes mitochondrial fission, while phosphorylation at the Drp1-S637 site inhibits mitochondrial fission. The Drp1-pS616:Drp1-pS637 ratio determines mitochondrial fission activity [9]. Wang et al.study confirmed that in LMVECs, CS increases Drp1-pS616, decreases Drp1-pS637, and increases the Drp1-pS616:Drp1-pS637 ratio, thereby increasing mitochondrial fission activity [10]. Meanwhile, CS also increases the expression of FIS1, a receptor for Drp1-pS616 on the mitochondrial outer membrane, and decreases the expression of the mitochondrial fusion protein Mfn2 [10]. These results suggest that CS increases mitochondrial fission and decreases mitochondrial fusion in LMVECs, leading to endothelial barrier dysfunction and increasing the risk of acute respiratory distress syndrome. In addition, CS exposure can increase adenosine levels leading to ECs injury and emphysema disease [64]. Sustained adenosine exposure promotes intracellular adenosine uptake via nucleoside transporter proteins, followed by activation of p38 and JNK in mitochondria, which ultimately leads to mitochondrial defects and activation of the mitochondria-mediated intrinsic apoptotic pathway, leading to apoptosis of lung ECs [64].

**Fig. 3:**
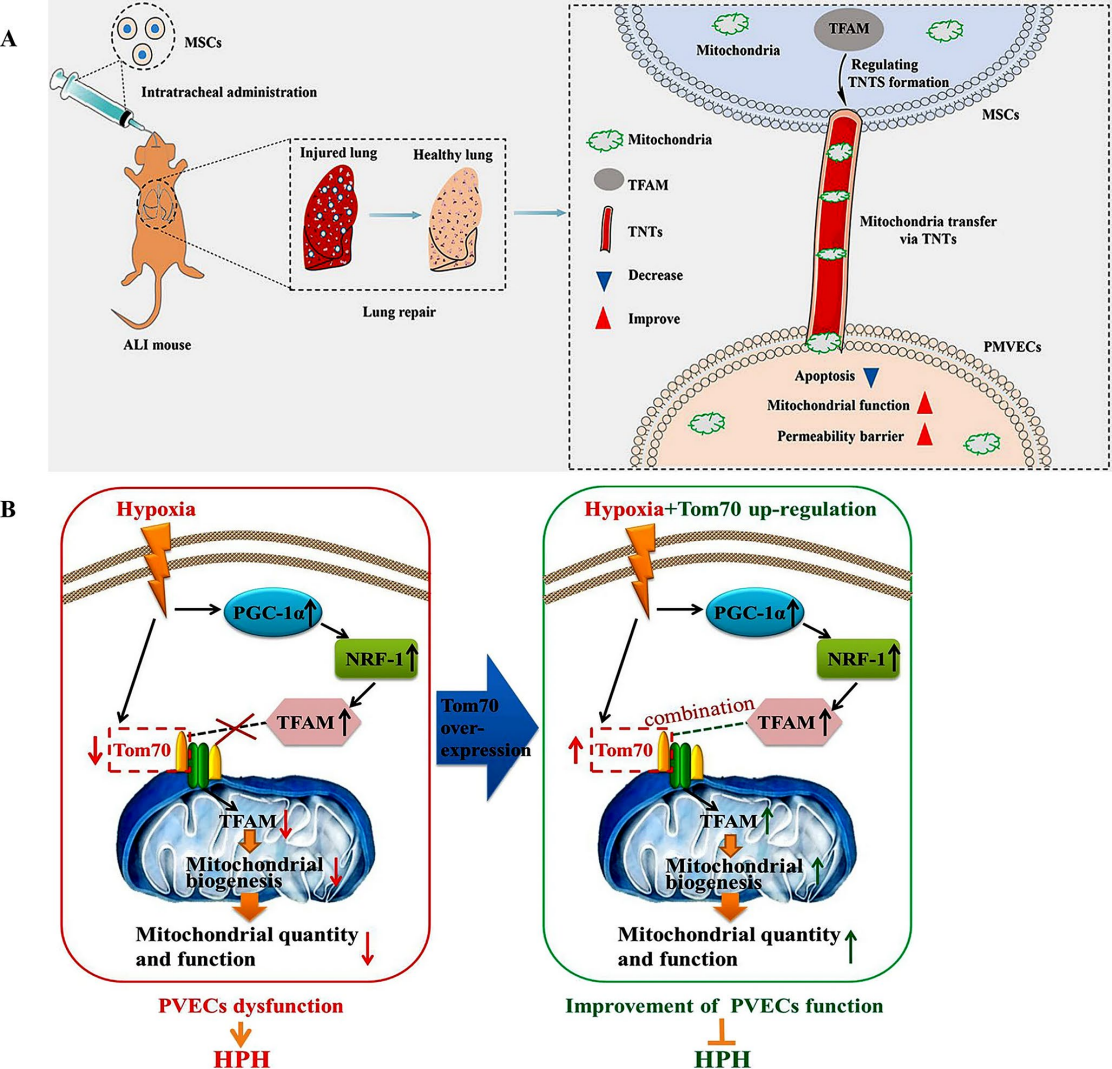
Diseases and mechanisms of lung endothelial injury caused by mitochondrial abnormalities. (**A**), Schematic model of regulation of mitochondrial transcription factor A (TFAM) expression in MSCs played a critical role to improve the permeability barrier of pulmonary microvascular endothelial cells (PMVECs) by tunneling nanotubes(TNTs) mediating mitochondrial transfer in sepsis-associated acute lung injury(ALI). Copyright 2023, Reprinted with from permission from Nature Publishing Group [72]. (**B**), Schematic illustrates that hypoxia induced the decreased expression of translocase of the outer mitochondrial membrane 70(Tom70) in pulmonary vascular endothelial cells (PVECs), reduced the mitochondrial biogenesis-associated TFAM protein transporting into mitochondria, inhibited mitochondrial biogenesis, caused PVECs dysfunction, and prompted the formation of hypoxic pulmonary hypertension(HPH). However, up-regulation of Tom70 abolished the hypoxia-induced injurious effects in PVECs and alleviated HPH. Copyright 2023, Reprinted with from permission from BioMed Central [66]

The concentration of oxygen is also an important factor influencing the function of pulmonary vascular ECs (PVECs), among which hypoxia is one of the main factors leading to dysfunction of PVECs. Ma et al. found upregulation of circKrt4 expression in hypoxic pulmonary arterial ECs. The increase in circKrt4 inhibits the cytoplasmic-mitochondrial shuttle of glycerol kinase (Glpk) bound to mitochondria, leading to mitochondrial dysfunction and further impairing pulmonary arterial ECs [65]. Hypoxia reduces the content of mitochondrial transcription factor A (TFAM) in mitochondria, inhibiting mitochondrial biogenesis and causing PVEC injury [66]. This mechanism is closely related to the translocase of the outer mitochondrial membrane (TOM) protein [66]. A crucial part of mitochondrial biogenesis is played by TOM, also referred to as the mitochondrial gate [67]. During mitochondrial biogenesis, precursor proteins synthesized in the cytosol need to be transported into the mitochondria via the TOM to exert their biological functions [68, 69]. Under hypoxic conditions, although the expression of mitochondrial biogenesis markers PGC-1α, NRF-1, and TFAM is increased, the expression of TOM70 in PVECs decreases, preventing the transport of TFAM into the mitochondria (Fig. 3B) [66]. This results in a reduction of TFAM within the mitochondria, thereby inhibiting mitochondrial biogenesis and causing dysfunction in PVECs. In contrast to hypoxia, Yao’s research suggests that a high-oxygen environment can also induce apoptosis of pulmonary ECs [70]. A high-oxygen environment reduces mitochondrial oxidative phosphorylation, leading to mitochondrial dysfunction and increased EC apoptosis [70]. Furthermore, excessive generation of ROS and mitochondrial damage exacerbate the inflammatory process of ALI. In terms of therapeutic strategies, the antioxidant mitoQ can reduce excessive mitochondrial ROS production through the Nrf2-MafF/ARE pathway, thereby improving mitochondrial function and alleviating lung inflammation [58]. Kong’s study indicates that the endogenous ligand, Apelin-13, for the seven-transmembrane G-protein-coupled receptor APJ can improve LPS-induced lung injury, pulmonary vascular permeability, and mitochondrial function in ALI mice, promoting autophagic flux [71]. The specific mechanism may involve Apelin-13 regulating mitochondrial function and autophagy by enhancing AMPK phosphorylation [71]. Zhang’s study indicates that mitochondrial transfer is the primary mechanism by which mesenchymal stem cells (MSCs) treat sepsis-related acute lung injury [72]. Specifically, TFAM in MSCs regulates the formation of tunneling nanotubes (TNTs), enabling the transfer of mitochondria from MSCs to PMVECs. This process reduces apoptosis in PMVECs, increases mitochondrial function and the permeability barrier in PMVECs, thereby mitigating sepsis-related acute lung injury (Fig. 3A).

### Kidney-related vascular diseases

**Fig. 4:**
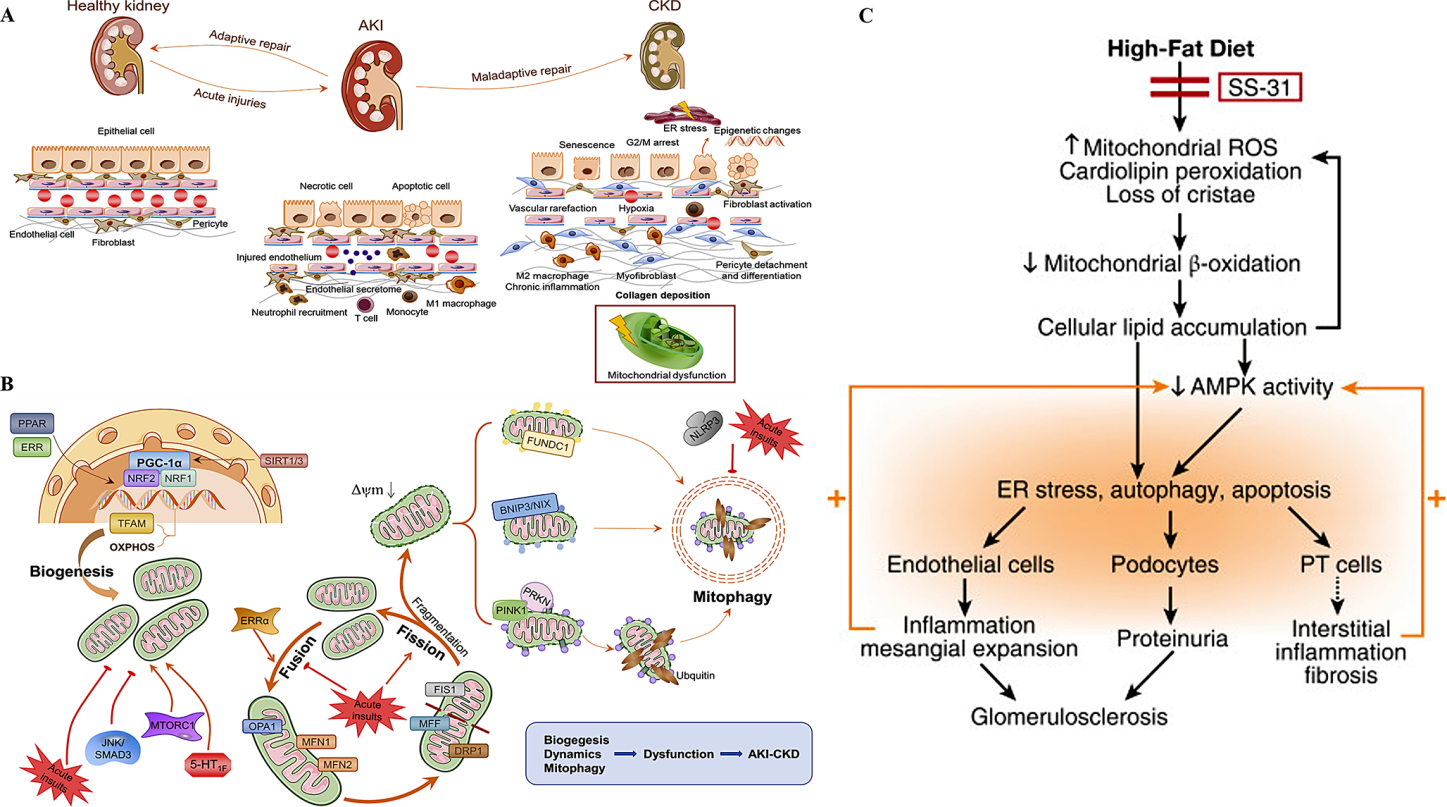
Diseases and mechanisms of renal endothelial damage caused by mitochondrial abnormality. (**A**), Schematic illustration of pathophysiological processes involved in the acute kidney injury (AKI)-chronic kidney disease (CKD) continuum mediated by mitochondrial damage [73]. (**B**), Impaired mitochondrial biogenesis (MB), mitochondrial dynamics, and mitophagy contribute to the transition from acute kidney injury (AKI) to chronic kidney disease (CKD). Copyright 2020, Reprinted with from permission from American Physiological Society [73]. (**C**), While high-fat diet (HFD) causes mitochondrial dysfunction and endothelial cell damage, leading to proteinuria, glomerular inflammation and glomerulosclerosis, and other kidney diseases, SS-31 is able to prevent almost all glomerular and tubular damage caused by HFD. Copyright 2016, Reprinted with from permission from Elsevier B.V [79]

Acute kidney injury (AKI) is widely recognized as a significant risk factor for the occurrence and progression of chronic kidney disease (CKD) [73]. The exact mechanisms by which a normal kidney transitions to acute kidney injury (AKI) and progresses from AKI to chronic kidney disease (CKD) are not yet fully understood, but several cellular and molecular mechanisms have been identified (Fig. 4A) [73]. Recent evidence suggests that mitochondrial biogenesis, mitochondrial dynamics, and mitophagy play critical roles in the progression from AKI to CKD (Fig. 4B) [73]. Considering that the mitochondria involved in the progression from AKI to CKD are specific to renal cells, this section will not elaborate further.

Early mitochondrial dysfunction in AKI is a key factor leading to tubular damage and persistent renal dysfunction [73]. Mitochondrial dynamics, maintained by fission and fusion, play a crucial role in preserving mitochondrial function [74]. In AKI, mitochondrial fission predominates in mitochondrial dynamics and is a major contributor to mitochondrial fragmentation [74]. Mitochondrial division and fragmentation in AKI pathogenesis are mediated by cellular sirtuins [74], with SIRT3 located in mitochondria being a major regulator of mitochondrial acetylation [75]. Research by Anna et al. indicates that Sirt3 deficiency, while not directly causing renal disease, promotes endothelial dysfunction and exacerbates kidney injury [75].

Additionally, renal I/R is one of the primary factors leading to AKI [76]. A study by Jankauskas et al. suggests that oxidative stress and endothelial mitochondrial damage are the main pathogenic factors of endothelial injury in renal I/R, and mitochondrial-targeted antioxidants can protect tissues from I/R effects [77]. Jankauskas et al. found significant endothelial mitochondrial damage after 40 min of ischemia followed by reperfusion [77]. Characteristics of endothelial mitochondrial damage include localized swelling of the mitochondrial matrix and a significant increase in cristae junction and intermembrane spaces, with a reduction or disappearance of cristae in some mitochondria. The mitochondrial-targeted antioxidant 10-(6′-plastoquinonyl) decylrhodamine 19 (SkQR19) can alleviate functional and morphological damage to renal vascular ECs after I/R [77]. Liu’s research suggests that SS-31 can protect EC mitochondria and reduce EC injury during renal ischemia [78]. SS-31 is a cell-permeable peptide that targets the inner mitochondrial membrane, selectively binds to cardiolipin, inhibits cardiolipin peroxidation, and significantly reduces loss of peritubular capillaries and cortical arterioles four weeks after ischemia, protecting proximal tubular cell mitochondria and reducing EC injury [78]. Additionally, under a high-fat diet (HFD), SS-31 can protect the normal mitochondrial structure of renal cells, block the vicious cycle of lipid accumulation caused by impaired mitochondrial fatty acid β-oxidation, improve renal AMPK activity, and prevent endoplasmic reticulum stress and apoptosis (Fig. 4C) [79].

In addition to AKI, the occurrence of renal vascular disease (RVD) is closely associated with mitochondrial damage. RVD is a significant cause of secondary hypertension and renal dysfunction, characterized by unilateral or bilateral renal artery stenosis (RAS) or occlusion [80]. Renal injury in RVD results from the combined effects of ischemia and metabolic abnormalities [80]. Ischemia is caused by inadequate perfusion due to RAS, while metabolic abnormalities are usually induced by metabolic syndrome (MetS). Previous studies have speculated that in RVD, the coexistence of MetS and renal artery stenosis may synergistically damage EC mitochondria, thereby exacerbating renal tissue injury [80]. Nargesi’s research suggests that metabolic syndrome (MetS) and RAS can damage pig renal artery EC mitochondria, impairing EC function [80].

### Cerebrovascular diseases

**Fig. 5:**
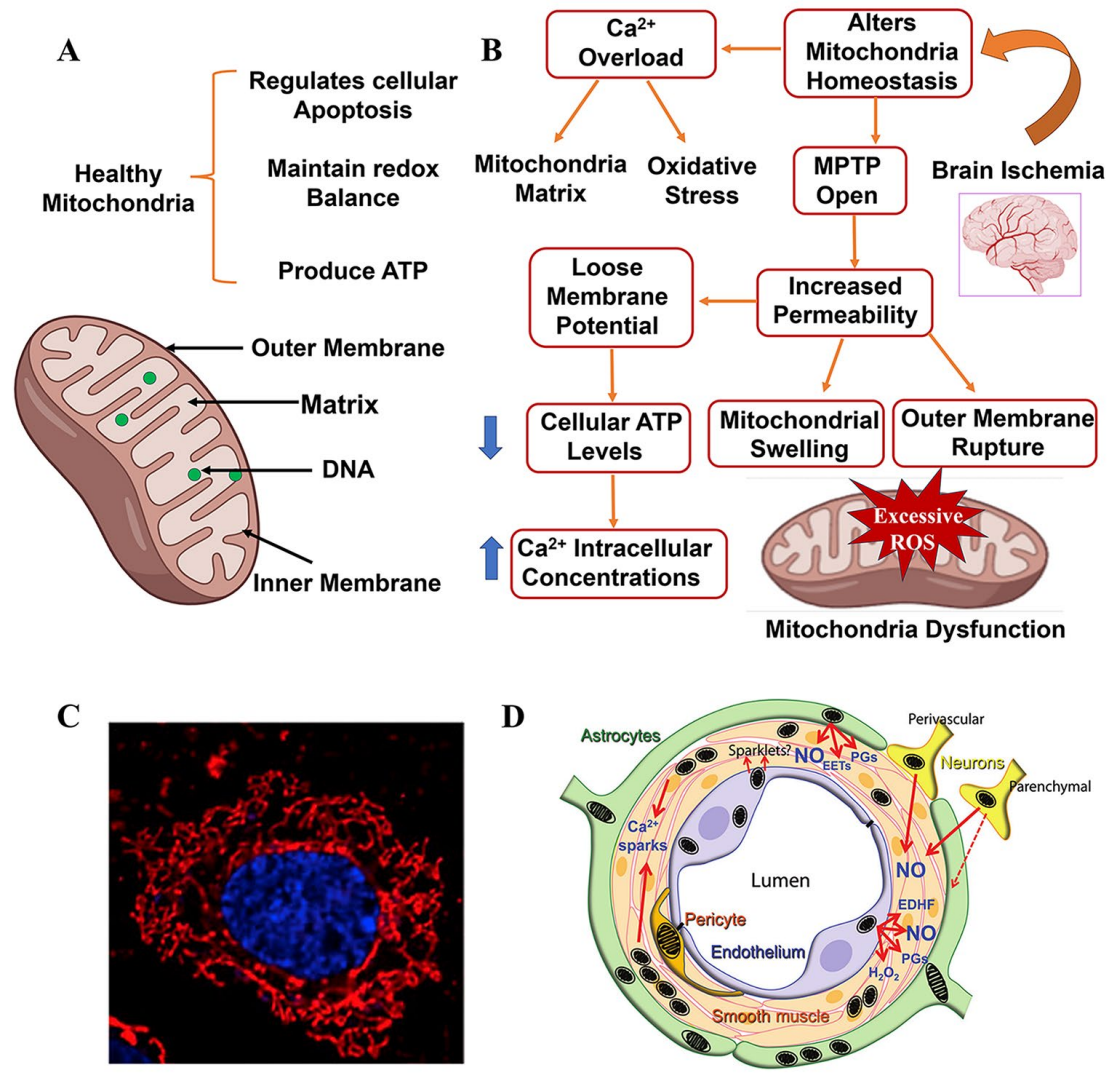
Mitochondrial abnormalities lead to endothelial damage in cerebral vessels and diabetic microvessels. Mitochondrial pathophysiology in brain ischemia. (**A**), The function and structure of healthy mitochondria. (**B**), The pathological process of mitochondria subjected to ischemia. (**C**), The morphology of mitochondria within cerebral vascular endothelial cell of live rat. Using mitochondrial stain and Hoechst nuclear stain to stain the mitochondria (red) and the nuclei (blue) in microvascular endothelial cells. Copyright 2014, Reprinted with from permission from Karger Publishers [94]. (**D**), Different cell types within neurovascular units of the brain could influence cerebrovascular tone through mitochondrial mechanisms, such as ischemia or cortical diffusion inhibition. Focal activation of neurons will directly affect mitochondria in these neurons and lead to NO production and increase of local blood flow. Copyright 2016, Reprinted with from permission from Wiley-VCH [82]

Mitochondria are the core regulators of vascular tone within the neurovascular unit (NVU) in the brain, where the interaction of mitochondria from various NVU cells in response to stimuli determines vascular tone, with endothelial cell mitochondria acting as environmental sensors (Fig. 5D) [81, 82]. Compared to other tissues and cells, the concentration of mitochondria in brain vascular ECs is higher than in ECs located elsewhere (Fig. 5C) [81]. Additionally, due to the high metabolic characteristics of the blood-brain barrier and its support for numerous transport systems, brain ECs possess an extensive mitochondrial network, with increased mitochondrial mass relative to other EC types(Fig. 5C) [83]. Mitochondrial function is closely associated with the physiological and pathophysiological processes of ECs, thus mitochondrial dysfunction may be a significant contributor to cerebrovascular pathologies [84, 85].Traumatic brain injury (TBI) is categorized into primary and secondary types [86]. Considering that the mechanisms of secondary injury are overly complex and current research has not identified definitive mechanisms, we will not discuss secondary brain injury [86]. Gerben’s research reveals that both TBI and cortical spreading depolarization (CSD) induce mitochondrial dysfunction, including impaired calcium homeostasis, decreased ATP production, and increased ROS generation [86]. Further observations using electron microscopy demonstrated morphological changes in blood vessels and mitochondrial cristae damage in astrocytes, pericytes, and ECs following TBI and CSD [86]. Based on these findings, Gerben suggests that mitochondrial damage induced by TBI and CSD may be associated with alterations in vascular morphology and cerebral blood flow (CBF) [86]. Studies have reported that exogenous brain mitochondrial transplantation can alleviate mitochondrial dysfunction in TBI [87]. In mouse models, exogenous mitochondrial intervention reduced cell apoptosis, promoted angiogenesis, alleviated brain edema, and mitigated blood-brain barrier leakage in TBI mice [87]. In vitro treatment with exogenous mitochondria in hypoxic cells improves cellular respiration control rates, increases oxygen consumption rates (OCR), and reverses significant losses in synaptic plasticity-related proteins [87]. Tight junction proteins (TJPs) are constituents of endothelial connections crucial for maintaining the integrity of the endothelial barrier [88]. Treatment with exogenous mitochondria significantly upregulates the expression levels of TJPs after mitochondrial therapy, thereby protecting the endothelial barrier [87]. These studies suggest that exogenous mitochondrial intervention is a potential new therapeutic strategy for TBI.

Mitochondrial dysfunction can also lead to vascular cognitive impairment (VCI), a form of dementia caused by cerebrovascular diseases, with a high-fat diet being one of its common triggers [89]. In vitro studies indicate that a high-fat environment causes swelling of human brain microvascular EC mitochondria and increases mitochondrial superoxide production, resulting in cerebrovascular endothelial lipotoxicity [89]. Mitochondrial dysfunction is not only a key pathogenic factor in vascular cognitive impairment but also one of the key pathogenic factors in neurodegenerative diseases. Aliev et al. used oxidative stress marker 8OHG immunogold labeling to reveal the presence of 8OHG-immunopositive gold particles in Alzheimer’s disease (AD)-affected vascular ECs and perivascular cells, suggesting a critical role of mitochondrial oxidative stress in the pathogenesis of vascular EC damage during AD progression [90]. With aging, mitochondria gradually lose their ability to support antioxidant function, becoming more prone to generating free radicals [91]. These free radicals not only cause excitotoxicity and neurodegeneration but also result in mitochondrial dysfunction [90, 91]. Mitochondrial dysfunction impairs ATP production, leading to further generation of free radicals, creating a vicious cycle [92]. Increased generation of free radicals induces the generation of β-amyloid peptide (Aβ) both in vivo and in vitro, thereby contributing to Alzheimer’s disease (AD) [91]. This may be a reason why mitochondrial dysfunction leads to the onset of neurodegenerative diseases.

Additionally, ischemic stroke is also closely associated with mitochondrial dysfunction. After the occurrence of cerebral ischemia, mitochondrial homeostasis is disrupted and a number of pathophysiological changes occur compared to healthy mitochondria (Fig. 5A, B). After stroke reperfusion, mitochondrial mass and mitochondrial DNA in the cerebral arteries of the stroke-affected hemisphere are maintained or enhanced, with diminished responses to other non-mitochondrial stimuli for dilation and constriction [82]. Further studies indicate that after hypoxic stress, not only ECs respond to mitochondria-induced postconditioning but also vascular exhibit maintained or enhanced responses to mitochondrial activators in cerebral vascular endothelium [93]. Busija proposes that targeted mitochondrial therapy can prevent further damage to endothelial and vascular smooth muscle cells, reducing brain injury [82].

The role of mitochondrial-derived ROS in cerebral vascular diseases remains controversial. Busija suggests that ROS released from mitochondria can protect the neurovascular unit, as mitochondrial activation following hypoxic or ischemic stress appears to protect cerebral vascular endothelium and promote blood flow restoration [94]. Conversely, Chrissobolis et al. argue that oxidative stress induced by mitochondrial-derived ROS is a major cause of endothelial dysfunction in cerebral circulation, reducing the bioavailability of NO and impairing endothelial maintenance of vascular tone [95]. EC injury can also lead to blood-brain barrier dysfunction. Radiation-induced brain injury (RIBI) is a common complication of brain tumor radiotherapy, characterized by blood-brain barrier dysfunction resulting from EC injury [96]. Current treatment strategies primarily focus on protecting ECs from oxidative stress damage. Research by Zhang et al. indicates that the fluorescent small molecule they developed, IR-780, can target mitochondrial regulation of oxidative stress in cerebral microvascular ECs, protecting cells from radiation damage [97]. Additionally, using SIRT1 to inhibit p66Shc transcription protects vascular ECs from oxidative damage [24].

### Diabetic microvascular disease

Mitochondrial dysfunction mediating endothelial injury can also lead to diabetic microvascular complications, a common cause of mortality and disability in diabetes, typically resulting from EC damage induced by hyperglycemia [98]. Studies on ECs isolated from diabetic patients have revealed that prolonged exposure to high glucose levels leads to mitochondrial swelling and subsequent endothelial edema [99]. Further investigation of EC mitochondria under high glucose exposure has shown manifestations of mitochondrial dynamics characterized by increased fission and inhibited fusion, resulting in mitochondrial fragmentation [100–102]. Upregulation of Drp1 expression and downregulation of Opa1 levels under high glucose conditions are major contributors to mitochondrial fragmentation [103]. A previous clinical study showed that EC damage in diabetic patients is closely associated with significantly impaired EC mitochondrial function [104].

A large body of evidence suggests that maintaining normal mitochondrial dynamics and activating mitochondrial autophagy improves mitochondrial function and EC resistance to high glucose [105–107]. Additionally, disruption of mitochondrial dynamics impairs mitochondrial metabolism, inhibits ATP synthesis, activates the JNK pathway, and induces mitochondrial-dependent apoptosis pathways [100]. Regarding mitochondrial autophagy, Xi found significantly reduced mitochondrial autophagic activity in high glucose-treated HUVECs [105]. Xi’s further study found that hyperglycemia significantly reduced the protein expression of mitochondrial autophagy-associated proteins, PINK1, Parkin, and Mfn2, in HUVECs [105]. Similarly, reduced mitochondrial autophagy was found in hyperglycemic human aortic ECs (HAEC), which Wang et al. found to be associated with reduced AMPK activity [108]. Conversely, overexpression of Sirt3 upregulates mitochondrial autophagy, enhancing AMPK/PGC1α expression, thereby preventing EC apoptosis. Interestingly, there exists a balance between mitochondrial fission and mitochondrial autophagy, where mitochondrial fragmentation induced by fission can promote mitochondrial autophagy, and fission has been reported as the initial signal for PINK1/parkin-mediated mitochondrial autophagy [109]. Activated mitochondrial autophagy, in turn, can eliminate fragmented mitochondria to counteract the harmful effects of fission, thereby preserving mitochondrial function. Mitochondrial autophagy in high glucose-treated rat aortic ECs has been found to normalize the ratio of phosphorylated Drp1 to total Drp1, selectively eliminating damaged mitochondrial subpopulations, leading to increased ATP production [110, 111]. Conversely, excessive mitochondrial autophagy can deplete mitochondria, leading to ATP exhaustion or deficiency and ultimately causing cell death [112].

In addition, diabetic microvascular complications are closely associated with mitochondrial oxidative stress, as evidenced by elevated oxidative stress induced by ROS in diabetic patients [113]. Mitochondrial respiratory dysfunction and NADPH oxidase (NOX) are two major sources of ROS in cells of diabetic patients [114], and oxidative stress plays a critical role in the development of cardiovascular diseases [115]. Research suggests that glycated or oxidized low-density lipoprotein (LDL) under diabetic conditions not only reduces mitochondrial oxygen consumption and membrane potential in ECs, but also impairs the activity of mitochondrial respiratory chain complex enzymes, leading to mitochondrial respiratory dysfunction [116, 117]. Moreover, diabetes-associated glycated LDL or oxidized LDL can increase NOX and ROS expression in ECs [114]. Therefore, Shen et al. propose that mitochondrial dysfunction and NOX activation resulting from metabolic disturbances associated with diabetes are the fundamental causes of cardiovascular diseases in diabetic patients [118]. The therapeutic drug empagliflozin inhibits mitochondrial fission through AMPK-dependent pathways, which in turn suppresses mitochondrial reactive oxygen species mtROS production and oxidative stress to protect the barrier function of CMECs [119]. This not only protects cardiac myocytes but also preserves cardiac microvascular ECs in diabetic patients. Currently, research targeting mitochondrial abnormalities and EC-related proteins as therapeutic targets for diabetic microvascular complications is gaining popularity. Mitochondrial dysfunction plays a central role in the occurrence and development of diabetic kidney disease (DKD). Therefore, targeting mitochondrial dysfunction in DKD may represent a novel therapeutic strategy for DKD patients. Additionally, ECs, as the main suppliers of lipids to glomerular cells, play an important role in lipid transport processes [120]. Vascular endothelial growth factor-B (VEGF-B) and mitochondrial proteins, which mediate this process, are considered therapeutic targets for systemic lipid toxicity mechanisms [121–123].

## Conclusion and perspectives

As both a mechanical and biological barrier for blood flow, ECs cover the inner layers of the heart, blood vessels, and lymphatic vessels with flattened endothelial tissue, acting as frontline defenders against vascular diseases. Dysfunction of ECs is closely associated with the occurrence of various diseases such as cardiovascular diseases, kidney diseases, lung-related diseases, cerebrovascular diseases, and diabetic microvascular diseases. Mitochondria play diverse roles in regulating intracellular calcium dynamics, ROS, and NO generation. Clinical trials and basic research have confirmed that mitochondrial pathway dysfunction is a key factor in stress-induced vascular endothelial injury. Moreover, as the primary site of aerobic cellular respiration, mitochondrial ROS production and dysfunction often determine the evolution of EC injury. Thus, mitochondrial abnormalities mediating vascular endothelial injury are considered crucial mechanisms in diseases such as cardiovascular diseases, kidney diseases, lung-related diseases, cerebrovascular diseases, and diabetic microvascular diseases.This article comprehensively reviews the role of mitochondrial abnormalities in vascular endothelial injury-related diseases and the corresponding research progress for treatments. The article discusses in detail the specific roles of mitochondria in different diseases, such as the relationship between air pollution-induced mitochondrial damage and cardiovascular diseases, as well as the role of mitochondrial dynamics and autophagy in diabetic microvascular complications. Additionally, the article explores potential drug strategies for treating these diseases, aiming to provide new insights and treatment approaches for clinical diagnosis of related vascular injuries. For example, the traditional Chinese medicine compound Xinmai’an tablets and isoliquiritigenin can protect the heart and vascular ECs by regulating mitochondrial function. Furthermore, mitochondrial-targeted antioxidants can protect tissues from I/R injury. In diabetic microvascular complications, high glucose-induced EC mitochondrial damage and dysfunction are key factors, suggesting that targeting mitochondrial dysfunction may represent a new therapeutic strategy.

Future research needs to further elucidate the specific molecular mechanisms underlying mitochondrial abnormalities and vascular endothelial injury, as well as how to prevent and treat cardiovascular diseases, kidney diseases, lung-related diseases, cerebrovascular diseases, and diabetic microvascular diseases by modulating mitochondrial function. In clinical treatment, with deeper understanding of mitochondrial biology, developing new treatment strategies, such as targeting mitochondrial fission and fusion proteins, regulating mitochondrial autophagy, and inhibiting ROS production, may provide new directions for treating vascular endothelial injury-related diseases. Additionally, the development of personalized medicine may make customized treatment of mitochondrial functional status possible for different patients. Furthermore, although some drugs have shown potential to protect mitochondria and ECs in experiments, more clinical trials are needed to verify the safety and efficacy of these drugs. Lastly, as the global aging trend intensifies, research on vascular endothelial injury and mitochondrial dysfunction in the elderly population will have significant social and clinical implications. Future research should focus on developing new preventive measures and treatment strategies to reduce the occurrence and progression of these diseases.

## Data Availability

No datasets were generated or analysed during the current study.
